# Image‐based assessment of natural killer cell activity against glioblastoma stem cells

**DOI:** 10.1002/2211-5463.13818

**Published:** 2024-05-13

**Authors:** Yuanning Du, Samuel Metcalfe, Shreya Akunapuram, Sugata Ghosh, Charles Spruck, Angela M. Richardson, Aaron A. Cohen‐Gadol, Jia Shen

**Affiliations:** ^1^ Medical Sciences Program Indiana University School of Medicine Bloomington IN USA; ^2^ Cell, Molecular and Cancer Biology Graduate Program Indiana University School of Medicine Bloomington IN USA; ^3^ Cancer Center Sanford Burnham Prebys Medical Discovery Institute La Jolla CA USA; ^4^ Department of Neurological Surgery Indiana University School of Medicine Indianapolis IN USA; ^5^ Department of Medical and Molecular Genetics Indiana University School of Medicine Indianapolis IN USA; ^6^ Indiana University Melvin and Bren Simon Comprehensive Cancer Center Indianapolis IN USA

**Keywords:** glioblastoma stem cells (GSCs), image‐based assays, immunotherapy, natural killer (NK) cells, NK cell migration and cytotoxicity

## Abstract

Glioblastoma (GBM) poses a significant challenge in oncology and stands as the most aggressive form of brain cancer. A primary contributor to its relentless nature is the stem‐like cancer cells, called glioblastoma stem cells (GSCs). GSCs have the capacity for self‐renewal and tumorigenesis, leading to frequent GBM recurrences and complicating treatment modalities. While natural killer (NK) cells exhibit potential in targeting and eliminating stem‐like cancer cells, their efficacy within the GBM microenvironment is limited due to constrained infiltration and function. To address this limitation, novel investigations focusing on boosting NK cell activity against GSCs are imperative. This study presents two streamlined image‐based assays assessing NK cell migration and cytotoxicity towards GSCs. It details protocols and explores the strengths and limitations of these methods. These assays could aid in identifying novel targets to enhance NK cell activity towards GSCs, facilitating the development of NK cell‐based immunotherapy for improved GBM treatment.

## Graphical abstract

Glioblastoma (GBM), fueled by glioblastoma stem cells (GSCs), poses a formidable challenge in oncology. Natural killer (NK) cells hold potential in targeting GSCs. This study introduces two image‐based assays evaluating NK cell migration and cytotoxicity against GSCs, facilitating the development of NK cell‐based immunotherapy for improved GBM treatment.

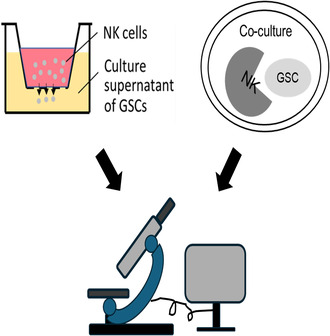

**Abbreviations**
ATCC: American Type Culture Collection
bFGF: basic fibroblast growth factor
DMEM: Dulbecco's Modified Eagle Medium
DMSO: dimethyl sulfoxide
DPBS: Dulbecco's Phosphate‐Buffered Saline
EGF: epidermal growth factor
FBS: fetal bovine serum
GBM: glioblastoma
GSCs: glioblastoma stem cells
H3K9: histone H3 Lys9
IL‐2: interleukin‐2
MHC‐I: major histocompatibility class I
NK cells: natural killer cells

Glioblastoma (GBM), the most frequent primary malignant brain tumor in adults, is known for its aggressiveness and lethality. The standard treatment protocol for GBM involves a combination of surgical intervention to remove as much of the tumor as feasible, followed by a course of radiotherapy and chemotherapy [1]. However, due to the recurrent nature of GBM, these treatments exhibit limited efficacy, resulting in notably low survival rates—reportedly less than 10% over a 5‐year period in the United States [1]. The resistance of GBM to standard treatments primarily arises from intratumoral heterogeneity, principally driven by glioblastoma stem cells (GSCs), which constitute a population of stem‐like cancer cells within the tumors [2]. These GSCs, possessing properties akin to normal stem cells such as self‐renewal and differentiation, play a pivotal role in GBM initiation, resistance to therapy, and the aggressive relapse of this form of brain cancer [3]. Unless the GSCs are eradicated, achieving a cure for GBM remains improbable.

The human immune system is divided into the innate and adaptive immunity, working together to protect against infections and cancers. The innate system, including natural killer (NK) cells and neutrophils, serves as the body's first line of defense, swiftly responding to potential threats [4, 5, 6, 7]. This system works alongside the adaptive immune system, including T cells and B cells, providing prolonged immunity through antibody responses and cell‐mediated immune responses. NK cells within the innate system can be activated via diverse receptors, including MHC‐I related (e.g., MICA or MICB) and non‐related (e.g., Nectin‐2 or CD155) receptors, enabling swift recognition and elimination of virus‐infected or tumor cells [8]. Previous studies demonstrate the capability of NK cells to recognize and destroy stem‐like cancer cells in various cancer types *in vitro* [9, 10]. However, in GBM, the immunosuppressive tumor microenvironment restricts NK cell function, impacting their infiltration and cytotoxicity [11, 12, 13]. Further exploration is needed to investigate the promising therapeutic potential of enhancing NK cell activity against GSCs within this environment for eradicating GSCs and treating GBM [14].

Based on this background, our study aims to develop two efficient image‐based assays evaluating NK cell migration and cytotoxicity towards GSCs. These assays provide sensitive, direct, and reproducible methods and could contribute to identifying new targets for boosting NK cell efficacy against GSCs. This could advance the development of NK cell‐based immunotherapy for enhanced GBM treatment.

## Materials

1. NK‐92MI (Cat # CRL‐2408; ATCC, Manassas, VA, USA)
2. MyeloCult H5100 culture media (Cat # 05150; STEMCELL Technologies, Vancouver, BC, Canada)
3. Hydrocortisone (Cat # 74142; STEMCELL Technologies)
4. GSC3565 (a gift from Dr Jeremy Rich, UPMC Hillman Cancer Center, Pittsburgh, PA, USA)
5. Neurobasal media (Cat # 12349015; Gibco, Billings, MT, USA)
6. B27 without vitamin A (Cat # 12587010; Gibco)
7. EGF (Cat # 236‐EG‐01M; R&D Systems, Minneapolis, MN, USA)
8. bFGF (Cat # 3718‐FB‐025; R&D Systems)
9. Sodium pyruvate (Cat # 11360070; Gibco)
10. Glutamax (Cat # 35050061; Gibco)
11. Penicillin/streptomycin (Cat # SV30010; Cytiva, Marlborough, MA, USA)
12. Lenti‐X‐293T cells (Cat # 632180; TaKaRa Bio, San Jose, CA, USA)
13. DMEM media (Cat # 11995065; Gibco)
14. FBS (Cat # 35015CV; Corning, Corning, NY, USA)
15. Accutase (Cat # AT104500; Innovative Cell Technologies, San Diego, CA, USA)
16. DPBS, no calcium, no magnesium (Cat #14190250; Gibco)
17. DMSO (Cat # BP2311; Fisher BioReagents, Pittsburgh, PA, USA)
18. Control and SUV39H1 shRNA vectors (gifts from Dr Charles Spruck, Sanford Burnham Prebys Medical Discovery Institute, La Jolla, CA, USA)
19. jetPRIME transfection reagent (Cat # 101000001; Polyplus, Illkirch‐Graffenstaden, France)
20. Transwell inserts with diameter 6.5 mm, pore size 5 mm (Cat # 3421; Corning)
21. Calcein AM (Cat # 65085339; eBioscience, San Diego, CA, USA)
22. U‐Shaped‐Bottom 96‐well plate (Cat # FB012932; Fisherbrand, Pittsburgh, PA, USA)
23. Hemocytometer (Cat # 304811; Weber Scientific, Hamilton, NJ, USA)
24. Axiovert 40 CFL Inverted Microscope (Zeiss, Oberkochen, Germany)
25. X‐Cite 120 Fluorescence Illumination Systems (EXFO Photonic Solutions Inc., Mississauga, Ontario, Canada)

## Methods

### Cell culture

Prepare the NK‐92MI cell culture media by adding 500 μL of 10 mm hydrocortisone and 5 mL of penicillin/streptomycin into 500 mL of MyeloCult H5100 culture media. Maintain the NK‐92MI cells expressing human IL‐2 in NK cell culture media in a 5% CO_2_, 37 °C incubator, passaging them every 2–3 days to retain a cell density between 2 × 10^5^ and 8 × 10^5^ cells·mL^−1^. For Lenti‐X‐293T cells, utilize DMEM media supplemented with 10% FBS for cell culture. GSC3565, a mesenchymal GSC model derived from a 32‐year‐old male GBM patient from University Hospitals, Cleveland by Dr Jeremy Rich lab, has been used in previous research [15, 16]. Regarding GSC3565 culture, employ Neurobasal media supplemented with B27 lacking vitamin A, 20 ng·mL^−1^ EGF, 20 ng·mL^−1^ bFGF, 1% sodium pyruvate, 1% GlutaMAX, and 1% penicillin/streptomycin [17].

### Lentivirus preparation, infection, and GSC conditioned media collection

The lentivirus packaging plasmids (pMD2.g and psPAX2) alongside control or SUV39H1 shRNA vectors are transfected into Lenti‐X‐293T cells cultured in DMEM media using jetPRIME transfection reagent. Following 24 h, the DMEM media is replaced with GSC culture media and incubated at 37 °C for an additional 48 h. The collected supernatant containing lentivirus undergoes filtration through 0.45 μm filters before GSC infection. To infect GSCs, incubate the lentivirus expressing either control or SUV39H1 shRNA with GSCs for 24 h, followed by a media change for another 48 h. Gather the conditioned media from these GSCs for NK cell migration assay. Simultaneously, portion the surplus conditioned media into 1.5 mL tubes and store them at −80 °C for future utilization. The GSCs transduced with either control or SUV39H1 shRNA are collected for GSC‐NK co‐culture and NK cell cytotoxicity assay.

### Image‐based NK cell migration assay

1. Precondition transwell inserts by introducing 600 μL of freshly prepared GSC media into the lower compartment of 24‐well plate wells, followed by the placement of inserts. Subsequently, add 100 μL of NK cell media into the upper compartment of the inserts. Incubate the setup for 1 h in a 5% CO_2_, 37 °C incubator.
2. Harvest NK‐92MI cells into a 15 mL centrifuge tube, subjecting them to centrifugation at 500 ***g*** for 5 min.
3. Aspirate the media and resuspend the cells in 1 mL of fresh NK cell media that has been pre‐warmed in a 37 °C water bath. Subsequently, quantify the NK‐92MI cells using a hemocytometer.
4. Retrieve the preconditioned 24‐well plate from the incubator. Remove 600 μL of media from the lower compartment of each well and replace it with 600 μL of GSC conditioned media collected when culturing GSCs transduced with control or SUV39H1 shRNA, as detailed in the ‘Lentivirus preparation, infection, and GSC conditioned media collection’ section.
5. Place the preconditioned transwell inserts into these wells and withdraw the initial 100 μL of NK cell media. Add 100 μL of 10^5^ NK‐92MI cells into each transwell insert.
6. Include fresh GSC media in lower compartment of 24‐well plate wells with NK‐92MI cells in the inserts, serving as negative controls.
7. Incubate the plate for 4 h in a 5% CO_2_, 37 °C incubator.
8. Extract the inserts and examine the lower compartment of the wells using a Zeiss Axiovert 40 CFL inverted microscope. Any migrated NK‐92MI cells should be observable in the wells.
9. Capture images of NK‐92MI cells in the wells using zen blue software connected to the microscope at 4× magnification. For each group, count the total number of NK‐92MI cells in each of the four selected fields before using graphpad prism (GraphPad Software, LLC., La Jolla, CA, USA) to generate the bar chart for comparison.

**Fig. 1:**
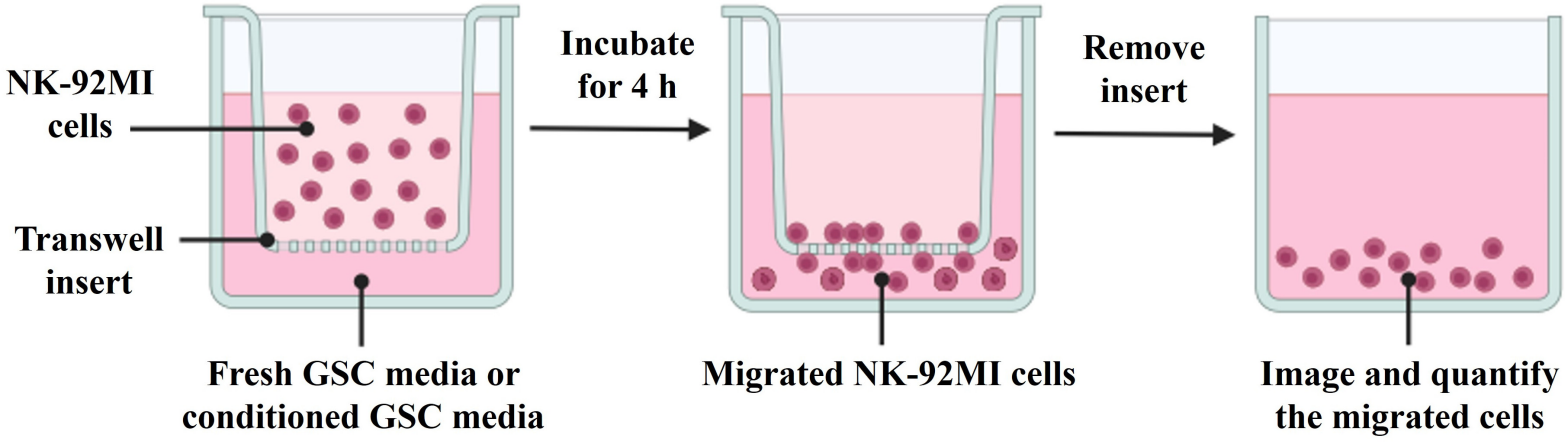
Schematic of the image‐based NK cell migration assay.

Please refer to the schematic of this assay in Fig. 1.

### Image‐based NK cell cytotoxicity assay

1. Centrifuge GSCs previously transduced with either control or SUV39H1 shRNA as detailed in the ‘Lentivirus preparation, infection, and GSC conditioned media collection’ section at 500 ***g*** for 5 min.
2. Resuspend the resulting cell pellets in 1 mL of 0.25% accutase and incubate at 37 °C for 5 min to obtain a single‐cell suspension.
3. Subject the cell suspension to another round of centrifugation at 500 ***g*** for 5 min. Resuspend the cells in 3 mL of GSC culture media that has been pre‐warmed in a 37 °C water bath.
4. Add 1.5 μL of calcein AM solution (10 mm stock in DMSO) to the cells to achieve a final concentration of 5 μm, and gently mix. Incubate the mixture for 30 min in a 5% CO_2_, 37 °C incubator.
5. Centrifuge the calcein AM‐labeled GSCs at 500 ***g*** for 5 min.
6. Wash the cells 3 times with 5 mL of DPBS to remove excess calcein AM dye.
7. Centrifuge NK‐92MI cells at 500 ***g*** for 5 min. Resuspend them in fresh NK cell media pre‐warmed in a 37 °C water bath.
8. Utilize a hemocytometer to count both the calcein AM‐labeled GSCs and the NK‐92MI cells.
9. Adjust the GSC concentration to 10^5^ cells·mL^−1^ in GSC culture media and the NK‐92MI cells to 2 × 10^6^ cells·mL^−1^ in NK cell media.
10. In a U‐Shaped‐Bottom 96‐well plate, place calcein AM‐labeled GSCs (10^4^ cells per 100 μL per well) with or without NK‐92MI cells (2 × 10^5^ cells per 100 μL per well) in duplicate. Utilize GSCs (control vs. SUV39H1 knockdown) only with 100 μL NK cell culture media as controls.
11. Incubate the cells for 4 h in a 5% CO_2_, 37 °C incubator.
12. Post‐incubation, capture fluorescence images for the calcein AM signal (green) in the wells using a fluorescence microscope (Axiovert 40 CFL Inverted Microscope) connected to X‐Cite 120 Fluorescence Illumination Systems at 10× magnification.
13. Quantify the calcein AM signal in the images. Calculate the percentage viability of GSCs using the formula: Viability % = (# of ‘green’ GSCs co‐cultured with NK‐92MI cells/# of ‘green’ GSCs in controls) × 100.

**Fig. 2:**
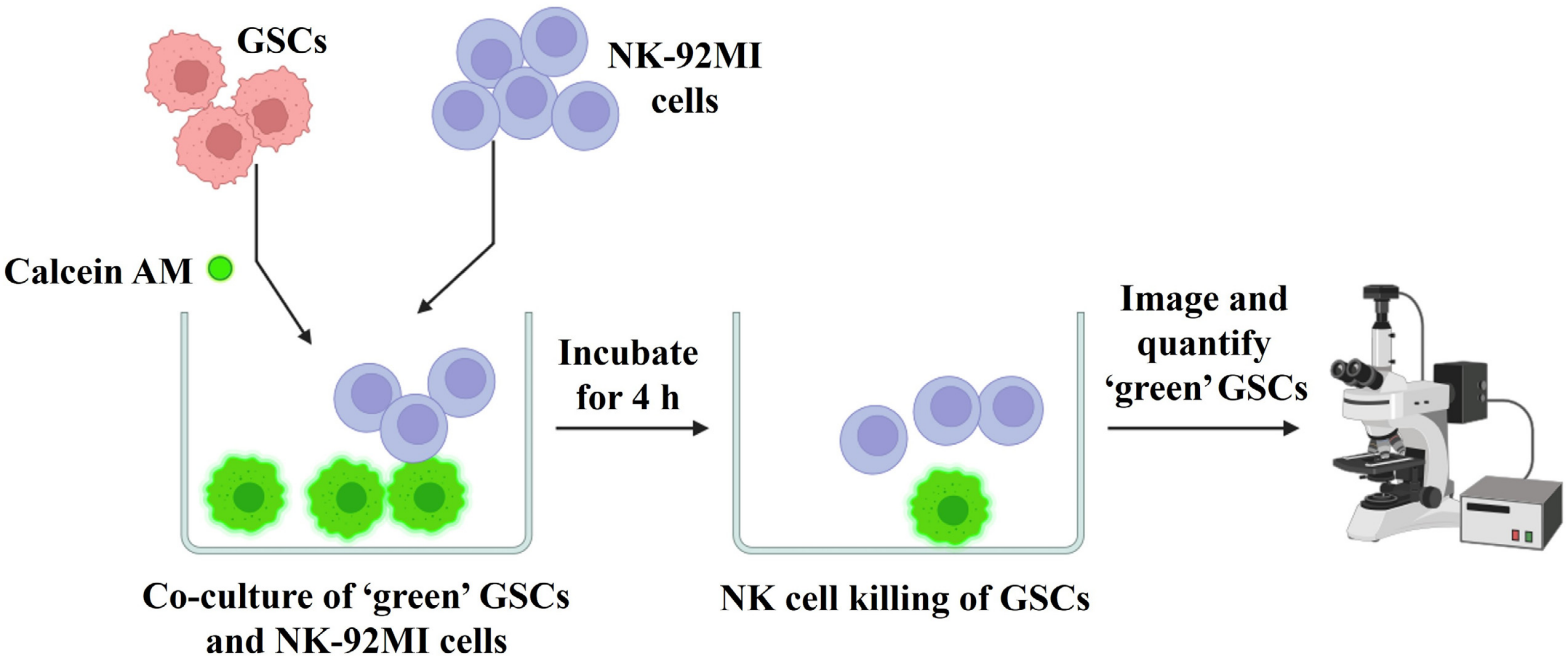
Schematic of the image‐based NK cell cytotoxicity assay.

Please refer to the schematic of this assay in Fig. 2.

## Results and Discussion

**Fig. 3:**
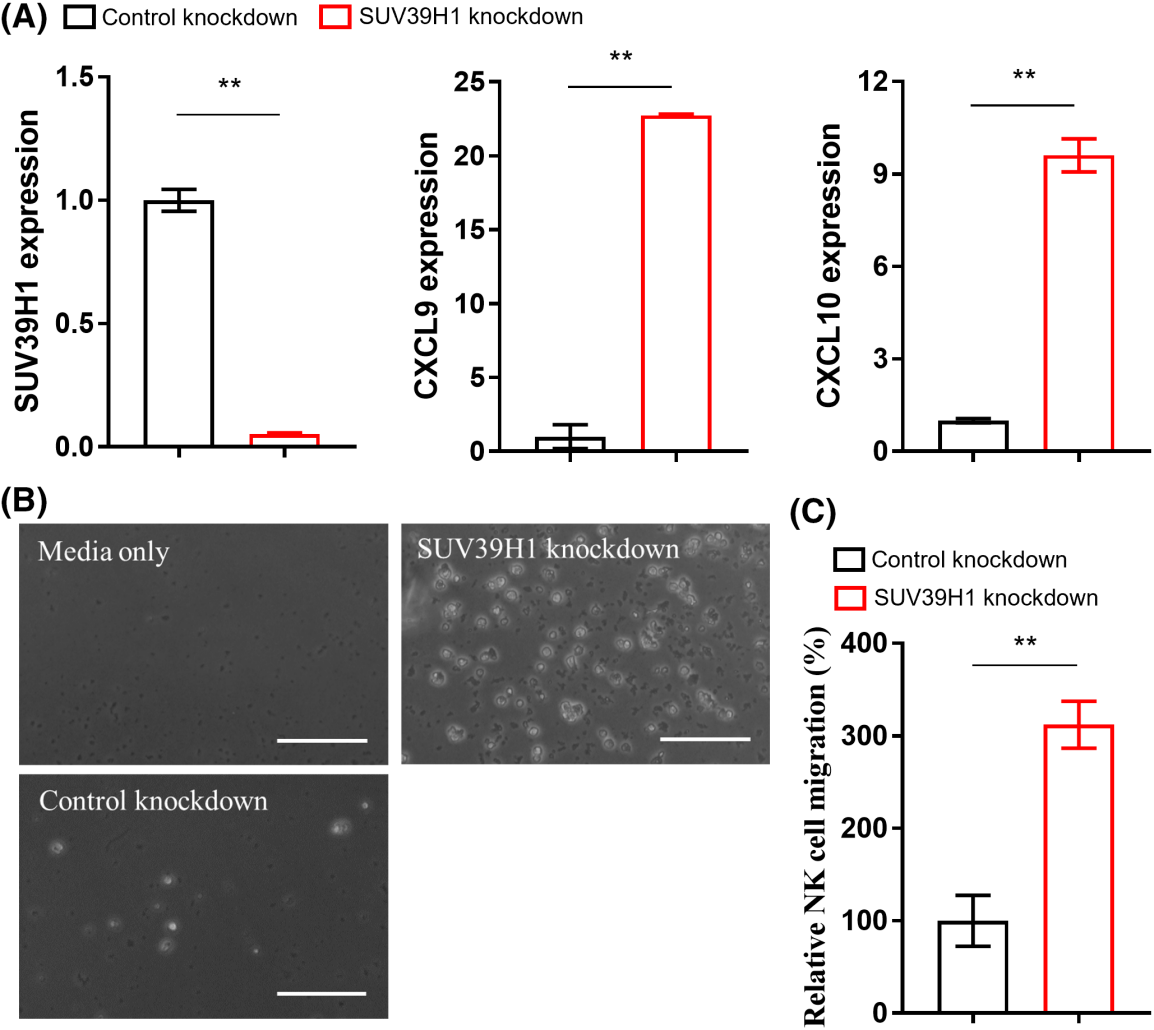
(A) qPCR detection of indicated gene expression in GSCs. Data represent mean ± SEM. **P < 0.01 by unpaired *t* test. (B) Representative images of the NK‐92MI cells that migrated into the lower compartment of the 24‐well plate. Scale bar, 100 μm. (C) Quantification of the migrated NK‐92MI cells in different fields (*n* = 4). Data represent mean ± SEM. **P < 0.01 by unpaired *t* test.

The *SUV39H1* gene encodes a methyltransferase enzyme primarily responsible for catalyzing methylation at the histone H3 Lys9 (H3K9) site, essential for maintaining heterochromatin structure by repressing repetitive sequences [18, 19]. Our previous research has demonstrated that targeting SUV39H1 pathway in various cancer cells leads to increased expression of specific cytokines known to attract NK cells, such as CXCL9 and CXCL10 [19]. This upregulation of CXCL9 and CXCL10 was also observed in GSCs following SUV39H1 knockdown (Fig. 3A). Consequently, the secretion of these cytokines into the GSC media is expected to promote NK cell migration in our migration assay. In the NK cell migration assay, three distinct groups were examined: a control group with fresh GSC culture media, a conditioned media group from GSCs infected by lentivirus expressing control shRNA, and an experimental group with conditioned media from GSCs exhibiting SUV39H1 knockdown. Quantification of migrated NK‐92MI cells was performed by analyzing images acquired from the lower compartment of the wells. The results indicate a significant increase in the migration of NK‐92MI cells towards the conditioned media from GSCs with SUV39H1 knockdown (Fig. 3B,C).

**Fig. 4:**
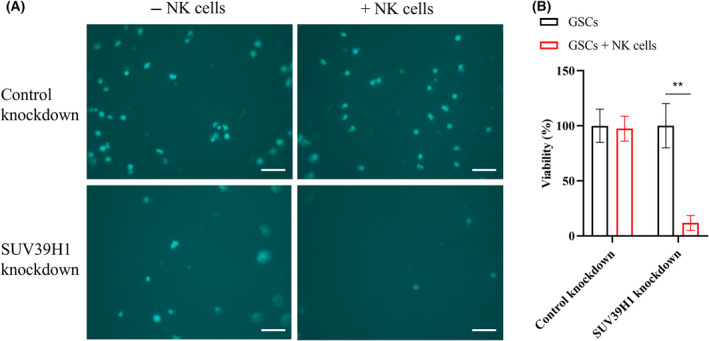
(A) Representative fluorescent images of calcein AM‐labeled GSCs. Scale bar, 100 μm. (B) Quantification of the viability of GSCs in different fields (*n* = 4). Data represent mean ± SEM. **P < 0.01 by two‐way ANOVA followed by Sidak's multiple comparisons test.

In the NK cell cytotoxicity assay, GSCs subjected to control conditions or SUV39H1 knockdown were initially labeled using calcein AM before their co‐culture with or without NK‐92MI cells. Calcein AM exhibits low cytotoxicity and easily permeates live cells, where it is hydrolyzed by intracellular esterases [20]. This process yields stable, vibrant green fluorescence, ideal for prolonged imaging without photobleaching. The results reveal a marginal decline in the viability of GSCs following co‐culture with NK‐92MI cells, suggesting limited cytotoxicity exerted by the NK cells against GSCs. However, the knockdown of SUV39H1 in the GSCs notably augmented the cytotoxic effects of NK‐92MI cells on the GSCs (Fig. 4A,B). Correspondingly, our previous study indicated that targeting the SUV39H1 pathway could increase the expression of several NK cell activating receptors on cancer cells, potentially leading to the recognition and subsequent elimination of these cancer cells by NK cells [19]. Consequently, these findings further validate the reliability and precision of our assay method.

Patient‐derived GSCs at an early passage were employed in the assays, underscoring the clinical significance and relevance of this research. This article delineates two image‐based methodologies aimed at efficiently examining NK cell migration and cytotoxicity towards GSCs. To quantify the migrated NK cells, unlike traditional approaches such as flow cytometry, which require expensive equipment and specialized training [21, 22, 23], our detection method is straightforward, cost‐effective, and adaptable to basic microscopy available in most laboratory setups, reducing operational complexities. Traditionally, the chromium release assay has been a mainstay for evaluating NK cell cytotoxicity [21, 24]. However, its reliance on radioisotopes like chromium‐51 presents health risks, demands specific training, and involves handling expensive and potentially hazardous radioactive materials. Furthermore, the assay's dependency on spontaneous release may yield inconsistent results. In contrast, our outlined protocol offers a simplified, easily implementable alternative applicable to laboratories equipped with a fluorescent microscope. Moreover, both of our assays enable direct quantification of cells (NK‐92MI cells in the migration assay and GSCs in the cytotoxicity assay) within the experimental wells, obviating the need for transfer or additional instrumentation. This not only saves time but also minimizes potential variations introduced during such additional processing steps. Additionally, it is important to acknowledge the limitations here. While aiding in comprehending NK cell behavior towards GSCs, these assays may not entirely mirror the complexity of *in vivo* settings. For instance, our use of single‐cell suspensions of GSCs in co‐culture assays with NK cells may not fully capture the *in vivo* scenario where NK cells penetrate the tumor. Therefore, utilizing 3D GSC neurospheres could better emulate the *in vivo* environment. Galat et al. recently employed GSC neurospheres to assess NK cell function [25], though ensuring uniform sphere sizes at the outset, especially for high‐throughput screening, is crucial yet challenging for enhancing assay accuracy. In addition, the influence of other immune cell players, such as T cells or macrophages, on the effects exerted by NK cells against GSCs in GBM microenvironment remains a pertinent question. Consequently, validating the outcomes obtained *in vitro* through subsequent *in vivo* investigations is imperative. This strategic approach would bolster the practical applicability of the insights garnered from these assays.

Collectively, this research introduces methodologies that are straightforward, dependable, and adaptable for application in diverse laboratory environments, enabling the investigation of NK cell migration and cytotoxicity against GSCs. While acknowledging the study's limitations, the outlined methodology holds promise in delineating the feasibility of identifying novel targets and leveraging NK cell immunotherapy for targeting GSCs and treating GBM.

## Tips & Tricks

1. Establishing an initial equilibration phase for the transwell inserts is crucial for preserving the optimal functionality of NK cells in the migration assay. This process entails a stepwise addition of GSC media to the wells within a 24‐well plate, followed by the insertion of transwell inserts, and subsequent addition of NK cell media into the inserts. A recommended minimum incubation period of 1 h for the plate is advised to achieve the desired equilibrium.
2. The inserts are supplied in packs of 12, prearranged within a 24‐well plate. If fewer than 12 migrations are being conducted, it's possible to extract the required number of inserts, utilizing a standard 24‐well plate, and then reseal the remaining inserts in their container.
3. Employing U‐Shaped‐Bottom 96‐well plates for the cytotoxicity assay might offer an improved spatial proximity conducive to the interaction between NK‐92MI cells and GSCs compared to regular flat‐bottom plates.
4. To optimize calcein AM staining of GSCs, conducting a preliminary experiment that varies concentrations and incubation times is advisable.
5. The use of DPBS for washing GSCs subsequent to calcein AM staining is crucial to minimize nonspecific high background staining.
6. Given the distinct culture media requirements for NK‐92MI cells and GSCs, extended co‐culture periods have the potential to affect their cellular traits and behaviors during analyses. Therefore, it is advisable to minimize prolonged co‐culture durations to uphold the integrity of experiments and mitigate the likelihood of changes in cellular characteristics.

## Conflict of interest

The authors declare no conflict of interest.

## Author contributions

YD, SM, SG, and JS performed the experiments. YD, SM, SA, CS, AMR, AAC‐G and JS contributed to writing, reviewing, and editing the manuscript. JS conceived and designed the project.

## Data Availability

The data that support the findings of this study are available from the corresponding author upon reasonable request.
